# Foliar *Epichloë gansuensis* Endophyte and Root-Originated *Bacillus subtilis* LZU7 Increases Biomass Accumulation and Synergistically Improve Nitrogen Fixation in *Achnatherum inebrians*

**DOI:** 10.3390/jof11070466

**Published:** 2025-06-20

**Authors:** Yuanyuan Jin, Zhenjiang Chen, Kamran Malik, Chunjie Li

**Affiliations:** State Key Laboratory of Herbage Improvement and Grassland Agro-Ecosystems, Key Laboratory of Grassland Livestock Industry Innovation, Ministry of Agriculture and Rural Affairs, Engineering Research Center of Grassland Industry, Ministry of Education, Gansu Tech Innovation Centre of Western China Grassland Industry, Centre for Grassland Microbiome, College of Pastoral Agriculture Science and Technology, Lanzhou University, Lanzhou 730000, China; jinyy@lzu.edu.cn (Y.J.); malik@lzu.edu.cn (K.M.)

**Keywords:** *Achnatherum inebrians*, *Epichloë gansuensis* endophyte, *Bacillus subtilis*, nitrogen, colonization, interactions

## Abstract

Although drunken horse grass (*Achnatherum inebrians*) can be simultaneously infected by the foliar endophyte *Epichloë gansuensis* and colonized by *Bacillus subtilis*, it remains unclear whether *Epichloë* endophyte symbiosis influences *B. subtilis* colonization, as well as how their interaction affects nitrogen fixation and assimilation. The purpose of the present study was to investigate whether *E. gansuensis* endophyte infection facilitates the colonization of *B. subtilis* in the roots of host plants, with a focus on understanding the interaction effects of the *E. gansuensis* endophyte and *B. subtilis* on plant growth and nutrient absorption. In this study, we measured the colony growth rate of *B. subtilis* LZU7 when co-cultured with *E. gansuensis* strains. In addition to an in vitro test, we investigated the root colonization of *Epichloë* endophyte-infected plants (E+) and *Epichloë* endophyte-free plants (E−) with the GFP-tagged *B. subtilis* LZU7 in an inoculation test. Furthermore, we evaluated the interactions between *E. gansuensis* endophyte symbiosis and *B. subtilis* LZU7 colonization on the dry weight, nitrogen fixation, nitrogen converting-enzyme activity, and nutrients for E+ and E− plants by labeling with ^15^N_2_. The results showed that the growth rates of *B. subtilis* LZU7 were altered and increased in a co-culture with the *E. gansuensis* endophyte. A significantly greater colonization of GFP-tagged *B. subtilis* LZU7 was detected in the roots of E+ plants compared with the roots of E− plants, suggesting that *E. gansuensis* endophyte symbiosis enhances the colonization of beneficial microorganisms. The combination of *E. gansuensis* endophyte symbiosis and *B. subtilis* LZU7 inoculation significantly altered the expression of the nitrogenase (*nifH*) gene, thereby promoting increased biological nitrogen fixation (BNF). The *E. gansuensis* endophyte infection and inoculation with *B. subtilis* LZU7 significantly increased δ15NAir in plants. Co-inoculation with the *E. gansuensis* endophyte and *B. subtilis* LZU7 significantly elevated NH_4_^+^ accumulation in the roots, depleted the NH_4_^+^ availability in the surrounding soil, and showed no measurable impact on the foliar NH_4_^+^ content. The observed alterations in the NH_4_^+^ content were linked to nitrogen-fixing microorganisms that promoted nitrogen fixation, thereby enhancing nitrogen uptake and contributing to greater biomass production in *A. inebrians*. Our findings highlighted the fact that a foliar symbiosis with the *E. gansuensis* endophyte enhances the recruitment of beneficial bacteria, and that the resulting interaction significantly impacts nitrogen fixation, assimilation, and allocation in host plants.

## 1. Introduction

Mutualistic interactions with symbionts are being increasingly recognized for the important roles they play in ameliorating stressors and facilitating the ecosystem services that their host organisms provide [1,2]. Such symbioses influence various processes in host plants, ranging from nutrient acquisition to interactions with pollinators, seed dispersers, and the biotic and abiotic environment [3,4,5]. Microorganisms are among the most important mutualists of plants. One group of important microbial symbionts that often interact mutualistically with their hosts comprises *Epichloë*, a fungal endophyte of grasses [6]. These fungi are asexual, are vertically transmitted, and live asymptomatically within the plant tissues of 20–30% of grass species worldwide, including many economically important forage and turf grasses [7]. Endophyte symbioses with grasses can increase host resistance to insect pests through the production of alkaloids [8], increase N absorption [9,10], enhance drought and salt tolerance [11,12,13], and confer protection from super-infections caused by fungal pathogens [14]. Consequently, these symbiotic relationships have been widely exploited in the breeding of resistant varieties, ecological restoration, and grassland improvement [15,16]. Much is known about the enhanced stress tolerance conferred by endophyte symbiosis; despite this, little is known about whether there are foliar endophyte infections that interact with other root beneficial microbes in these processes [17].

Root-associated microbiomes have been recognized as the “second genome” of plants, especially plant growth-promoting bacteria, which communicate and participate in interplay to increase nitrogen fixation, provide mineralizing nutrients, modulate the plant hormonal balance, and defend against pathogens [18,19,20,21]. For instance, *Bacillus* species are major industrial workhorse microorganisms and have been widely used in agriculture. They provide important services to the plant, such as preventing pathogen infections [22] and enhancing the colonization and N_2_ fixation of rhizobia in roots [23,24], thus promoting the yield of the associated plants [25]. *B. subtilis* is the best-characterized member of the genus *Bacillus*, which comprises Gram-positive bacteria that can modulate microbial interactions to regulate N transport and assimilation, thus improving N efficiency and sustainable agricultural development [26].

Nitrogen is a major growth-limiting nutrient for plant defense and growth as well as net primary productivity in terrestrial ecosystems, and the vast majority of the nitrogen (78%) in the atmosphere exists in the form of a single molecule, which is not directly available to plants [27]. Specific beneficial microbe taxas (e.g., nitrogen-fixing bacteria) are able to convert N_2_ into plant-available forms via nitrogen nitrogenase [28]. An endophyte infection in plants can not only modify these microbial community structures by mediating root exudates and plant litter but also reduce the N loss from the soil and improve the nitrogen use efficiency in host plants through these microbes [29]. Microbial community structure and composition are usually influenced by changes in N availability [10,30,31]. Endophyte infection induces host plants to use N for the synthesis of their characteristic bioprotective alkaloids, establishing N as an important currency of mutualism in vertically transmitted foliar *Epichloë* endophyte symbioses [32,33]. This positive feedback between the *Epichloë* endophyte and N is expected to stimulate N fixation by cycling microorganisms, thereby increasing N bioavailability and plant growth. The magnitude of these effects depends on the feedback strength toward microbes, which varies with different endophytic fungal symbionts [10,34]. To date, the ecological mechanisms explaining how *Epichloë* endophyte infections in grasses contribute to nutrient cycling and plant growth through interactions with beneficial microbes (*Bacillus* sp.) have been seldom explored [35]. Given this background, we hypothesized that in *A. inebrians*, an *E. gansuensis* endophyte infection would have a strong impact on nitrogen fixation and plant productivity by affecting the colonization of *B. subtilis* in the roots.

To test this hypothesis, we isolated and characterized the specificity and function of root endophytic bacteria from low-NH_4_^+^ -treated *A. inebrians* and assessed the interactions between *E. gansuensis* endophyte strains and endosymbiotic bacteria strains. We also monitored the colonization of plant roots by the GFP-tagged *B. strains* LZU7 strain in the *Epichloë* endophyte-infected (E+) plants relative to the *Epichloë* endophyte-free (E−) plants. To test for an effect of the interactions between the *E. gansuensis* endophyte infection and *B. subtilis* LZU7 inoculation on plant growth and nutrient absorption, we measured a number of indicators involved in plant growth as well as nitrogen fixation, transformation, and accumulation. Furthermore, we used models to quantify the contribution of the endophyte infection and *B. subtilis* LZU7 inoculation to plant nutrient accumulation and productivity.

## 2. Methods and Materials

### 2.1. Plant Materials

Different ecotypes of drunken horse grass (*A. inebrians*) were selected for this study based on their contrasting rhizosphere nitrogen transformation capacity, as previously described [34]. Briefly, a screening experiment was performed on 5 ecotypes by assessing their rhizosphere nitrogen transformation capacity, using the copy number of the nitrogen-cycle-related genes (i.e., *amoA*-AOB, *amoA*-AOA, *nirS*, *nirK,* and *nosZ*) [34]. The *A. inebrians* from Tianzhu in Gansu Province, China, was chosen to study its endosymbiotic bacteria and the interactions with the *Epichloë* endophyte on host nitrogen uptake and growth.

### 2.2. Detection and Isolation of E. gansuensis Endophyte

Sequencing and a comparison with the National Center for Biotechnology Information (NCBI) identified that the *A. inebrians* from Tianzhu was infected with the *E. gansuensis* endophyte [36]. *Epichloë* endophyte-infected (E+) and *Epichloë* endophyte-free (E−) *A. inebrians* were space-planted in field plots at 2 locations in Gansu, China, in 2017. E+ and E− seeds were harvested in each plot over of a 6-year study (2017–2023) and examined for endophyte hyphae through seed staining. Microscopic detection procedures and PCR analyses using the following *Epichloë*-specific PCR primer pair: tub2-exon 1d-1: GAGAAAATGCGTGAGATTGT, tub2-exon 4u-2: GTTTCGTCCGAGTTCTCGAC. The E+ and E− seeds used for fungal endophyte isolation in this study were collected during July 2023, and stored at 4 °C to maintain endophyte viability.

After peeling, the E+ seeds were surface-sterilized with 75% ethanol for 30 s and 1.0% NaOCl for 1 min, followed by a sterile water rinse 4–5 times. They were then dried on sterile filter paper and stored at 4 °C until use. To evaluate the sterilization effect, the final rinse water was dripped into Luria–Bertani medium (5 g of yeast extract, 10 g of tryptone, 10 g of NaCl, 18 g of agar, 1000 mL, and a pH of 7.0) and trypticase soy agar medium (15 g of pancreatic peptone, 5 g of soy peptone, 5 g of NaCl, 18 g of agar, 1000 mL, and a pH of 7.3 ± 0.2). After one week, no bacterial colonies were formed on the LB or TSA plates, indicating that the surface of the seeds was sterilized thoroughly. The sterilized seeds were placed on potato dextrose agar media (200 g of potato, 20 g of glucose, and 18 g of agar, with volumization to 1000 mL) using a sterilized tweezer. After a period of growth, the seedlings were directly cut using a sterilized scalpel, and then the incision was allowed to come into contact with the culture medium. The mycelium grew at the incision site after continued incubation. The purified fungus was obtained by inoculating the mycelium on new PDA media from 10 to 30 days after incubation, with 3 repetitions, and there were no other stray bacteria.

In order to determine whether the isolated fungal strain was the *E. gansuensis* endophyte, we carried out linear amplification and sequencing. In brief, fungal DNA was extracted using the D3195-01 HP Fungal DNA Mini Kit (Omega Biotek Inc., Norcross, GA, USA) according to the manufacturer’s directions. The fungal ITS2 region of the ribosomal DNA was amplified using the universal primers ITS1 and ITS4 (ITS1: TCCGTAGGTGAACCTGCGC, and ITS4: TCCTCCGCTTATTGATATGC) [37]. The PCR products were sequenced to obtain the internal transcribed spacer (ITS) sequence from the ribosomal DNA of the fungal strain. The amplified DNA sequence was analyzed using the Basic Local Alignment Search Tool (BLAST v2.16.0) to identify homologous sequences and assess the sequence similarity to existing sequences in the NCBI database (http://www.ncbi.nlm.nih.gov/).

### 2.3. Isolation and Identification of Endophytic Bacteria

The roots of the E+ plants treated with 0.01 mol/L ammonium nitrogen in the preliminary experiment were rinsed several times with sterile water to remove impurities attached to the surface of the roots [29], and they were then cut into shreds. Sterilization and an evaluation of the sterilizing effect were conducted in the same manner as in the above seed treatments. An amount of 500 mg of the root sample was accurately weighed and placed into a sterilized mortar. Sterilized quartz sand was added to disrupt and homogenize the roots sufficiently by grinding. After grinding, 5 mL of a 0.9% NaCl solution was added and the mixture was transferred to a 50 mL sterile conical centrifuge tube, shaken well, and sonicated for 1 min with occasional shaking. An amount of 1 mL of the supernatant was taken and extracting solutions were made with concentrations of 10^−6^, 10^−7^, and 10^−8^. A total of 100 μL of each concentration was spread onto LB agar plates, with three parallel replicates for each concentration. The Petri dishes were incubated in a dark incubator at 25 °C for 3–5 d. The endosymbiotic bacteria were isolated and purified by the plant tissue culture and scribing method. Briefly, a small amount of a bacterial colony was transferred aseptically using a pick-up loop to an LB solid medium for district zoning. To obtain more independently distributed individual cells, the colony was diluted using the point-to-line method. To clarify the morphological characteristics of the isolated strains, isolated pure-culture endophytic bacteria were stained using a Gram-stain kit (Beso Biotech, Zhuhai, China). The bacterial strains were coated onto a slide and fixed by heating over an alcohol lamp. The four steps of pre-staining, mordanting, decoloration, and re-staining were carried out. After each step, the slide was washed with water, blotted up, and covered with a glass sheet. The images were taken using an Olympus CX22 LED microscope (Shinjuku, Japan). Gram-positive bacteria were stained purple, while Gram-negative bacteria were stained red. The shape, size, surface, and color of the bacterial strains were observed with the naked eye.

The total DNAs was extracted from samples of the pure bacterial cultures with an Ezup Column Bacteria Genomic DNA Purification Kit (Sangon Biotech, Shanghai, China) by following the manufacturer’s instructions. The DNA concentration and purity were measured using a NanoDropND-1000 spectrophotometer (Thermo Fisher Scientific, Waltham, MA, USA). The 16S rRNA gene of the bacterial strains was amplified using the universal primers 27F (5′-GAGTTTGATCATGGCTCAG-3′) and 1492R (5′-GGTTACCTTGTTACGATC-3′). Each DNA template was amplified in triplicate in a 25 μL reaction mixture containing primers. The PCR conditions were as follows: 12.5 µL of 2 × EasyTaq PCR SuperMix (Sangon Biotech, Shanghai, China), 1.0 µL of forward primers, 1.0 µL of barcoded reverse primers, 1.0 µL of template DNA, and 9.5 µL of ddH_2_O. The PCR amplification program was as follows: 95 °C for 5 min; 30 cycles of 95 °C for 60 s, 52.5 °C for 60 s, and 72 °C for 2 min; and 72 °C for 10 min. The PCR product was stored at 4 °C until use. The PCR products were purified and sent to Sangon Biotech for double-end sequencing and splicing. A homologous comparison was performed between nucleotide sequences of the 16S rRNA gene in the sequenced strains and published sequences in the NCBI database. The phylogenetic tree was constructed with the MEGA7 software (v7.0.14) using the neighbor joining method with 1000 bootstrap values and was visualized using FigTree. v.1.4.2 [37].

### 2.4. Functional Characterization of Endophytic Bacteria

#### 2.4.1. IAA Determination

Single colonies from the plates were picked and added to LB broth (10 g of tryptone, 5 g of yeast, 10 g of NaCl, 1 L of distilled water, and pH 7.2) containing 5 mmol·L^−1^ of L-tryptophan, and the control group was without the addition of bacterial colonies. The bacteria were incubated in a shaker at 120 r.min^−1^ and 28 °C for 5 d. After the incubation period, 1 mL of the bacterial solution was put into a 1.5 mL centrifuge tube and centrifuged (12,000 rpm, 5 min) to obtain the supernatant. A total of 200 μL of supernatant was aspirated and mixed with 200 μL of Salkowski’s color development solution (50 mL of 35% HClO_4_ ± 1 mL of 0.5 mol·L^−1^ FeCl_3_), and the color change of the solution was observed after avoiding light for 30 min. A standard solution of IAA was used as the control. Then, 200 μL of the color reaction mixture was transferred into a 96-well plate, and three technical replicates were used to obtain an OD reading on a microplate reader at OD_530_. The IAA content of the strains was computed using the standard curve method.

#### 2.4.2. The Measurement of Siderophores

Single colonies from the plates were inoculated onto sterilized MKB medium (15 mL of glycerin, 3.28 g of FeCl_3_·6H_2_O, 5 g of acid hydrolyzed casein, 2.5 g ofMgSO_4_·7H_2_O, and 18 g of agar powder) and incubated for 2 d in an incubator at 28 °C in an inverted position. After 2 d, 10 mL of a CAS test solution (0.06 g/L chrome azurol sulphonate, 0.073 g/L hexadecyl trimethyl-ammonium bromide, and 0.0027 g/L FeCl_3_.6H_2_O) (60 °C) was added to each inoculated MKB plate; the color change of each plate was observed after 1 h.

#### 2.4.3. Determination of Nitrogen Fixation Potential

The isolated strains were inoculated into Ashby’s nitrogen-free medium (10 g of sugar, 0.2 g of KH_2_PO_4_, 0.2 g of MgSO_4_·7H_2_O, 0.2 g of NaCl, 0.1 g of CaSO_4_·2H_2_O, 5 g of CaCO_3_, 15 g of agar, 1000 mL, and a pH of 7.0–7.2) for inverted incubation at 28 °C for 4–7 d. If the bacterial strain had a nitrogen-fixation ability, the colonies were grown on a nitrogen-free medium.

#### 2.4.4. Real-Time Amplification Assay of *nifH*

To investigate whether the isolated bacterial strains were nitrogen-fixing, the *nifH* gene was detected. The *nifH* gene of the bacterial strains was amplified using the primers *nifH*F (5′-AAAGGYGGWATCGGYAARTCCACCAC-3′) and *nifH*R (5’-TTGTTSGCSGCRTACATSGCCATCAT-3’) [38]. Each DNA template was amplified in triplicate in a 25 μL reaction system (2.5 μL of 2 × Taq Master Mix1, 8.5 μL of ddH_2_O, 2.0 µL of DNA,1.0 µL of forward primers, and 1.0 µL of barcoded reverse primers). The PCR amplification program was as follows: 98 °C for 2 min; 30 cycles of 98 °C for 15 s, 55 °C for 30 s, and 72 °C for 30 s; and 72 °C for 5 min. The amplified PCR products were detected using 2% agarose gel electrophoresis.

### 2.5. Plate Confrontation Experiments Between Epichloë Endophyte and Endophytic Bacterial Strains

The plate confrontation experiments were conducted as follows: To assess the interactions between the *Epichloë* endophyte and endosymbiotic bacterial strains, 6 mm *E. gansuensis* endophyte cakes were inoculated in solid YEDP medium, and then endophytic bacteria were inoculated around the *E. gansuensis* using the crisscross method. After incubating for another 7 d, the inhibitory effects on colony growth were observed. The double plate method was as follows: *E. gansuensis* endophyte cakes (6 mm) were cultured in a 500 mL flask containing 200 mL of liquid YEDP medium at 150 rpm for 10 d at 23 °C. The culture solution was filtered through a 0.22 μm membrane and then coated onto a nitrogen-free medium to solidify. YEDP liquid medium filtered through a 0.22 μm membrane was used as a blank control. A liquid with 200 μL 10^−5^ and 10^−6^ cells mL^−1^ (OD = 0.5, 600 nm) was coated onto a nitrogen-free medium containing the *E. gansuensis* endophyte to count the number of single colonies.

### 2.6. Colonization of A. inebrians Roots by GFP-Tagged Endophytic Bacteria

Soil sterilization was performed as follows: A 100 g sample of unplanted soil from Jinniu Mountain in Yuzhong, Gansu, China (pH of 8.2, 53 mg·g^−1^ of total nitrogen, 0.96 mg·g^−1^ of total phosphorus, 0.03 mg·g^−1^ of NO_3_^−^, and 0.03 mg·g^−1^ of NH_4_^+^) was transferred into a 250 mL serum vial, which was then consecutively sterilized twice by autoclaving at 121 °C for 20 min, and sterilized again after 24 h. After sterilization, the soil was cooled in an ultra-clean test bench, and its moisture content was kept at 60% of the maximum water holding capacity.

The glasshouse experiments were conducted as follows: E+ and E− seeds were surface-sterilized and then placed on sterilized nitrogen-free MS medium to germinate. After 14 d, E+ and E− seedlings that exhibited uniform growth were transplanted into an experimental set up with sterilized soil, with four plants in each plot and four replicates. The roots were completely encapsulated in the soil. The E+ and E− seedlings were cultivated in a greenhouse under long-day conditions (16 h photoperiod, 25 ± 2 °C, light/dark cycle, with a light intensity of approximately 600 μmol·m^−2^·s^−1^).

To verify the effects of the *E. gansuensis* endophyte on endophytic bacteria colonization, the endophytic bacteria were tagged with red fluorescent protein (GFP) (vector pBE2R-mRFP1). For inoculation, the GFP-tagged endophytic bacteria strain was activated in LB medium, and colonies with 10^−5^ cells·mL^−1^ (OD_600_ = 0.5) were incubated for 24 h. A total of 100 μL of the bacterial suspension was applied to the potted plants by adding it to the bottom of the container 3 days after transplanting, and 100 μL of liquid medium was used as a blank control. After 15 days, root samples from the E+ and E− plants were surface-sterilized with 70% ethanol, collected, and then sectioned. Thin sections were observed under a confocal laser scanning microscope (CLSM, Olympus FV3000 confocal microscope, Shinjuku, Japan) using a condition excitation wavelength of 552 nm.

### 2.7. ^15^N Isotope Dilution Method

To verify the nitrogen conversion efficiency (NCE) of the *Epichloë* endophyte and the endophytic bacteria in *A. inebrians*, N_2_ was labeled with ^15^N. The ^15^N_2_ (98%) was purchased from Shanghai Chemical Reagent Research Institute Co., Ltd., Shanghai, China. The experimental set up tube was plugged with rubber sealing and the mouth was sealed with a three-way valve to prevent the entry of microorganisms and air (Figure S1). The set-up was evacuated with a vacuum pump and cleaned using argon to remove N_2_, and then 78% ^15^N_2_, 21% O_2_, and 1% argon were passed through. The incubation was carried out at 25 °C and 70% humidity for 15 days. The plant and soil samples were harvested separately from each treatment. The N content and ^15^N enrichment of the plants and soil were determined using a flow injection analyzer (FIAstar 5000 Analyzer, Foss, Hilleroed, Denmark) and a stable isotope ratio mass spectrometer (Mat 253, Thermo Fisher Scientific, Waltham, MA, USA). Biological nitrogen fixation (BNF) was calculated using the following equations: BNF (ug·g^−1^ DW) = TN (ug·g^−1^ DW) × atom%15Nexcess (atom%^15^Nexcess = atom%^15^Nsample-atom%^15^Ncontrol).

### 2.8. Plant and Soil Nutrient Determination

The gases were collected from each treatment (four replicates) on day 15 and detected using gas chromatography (GC-2014C, Shimadzu Limited, Kyoto, Japan) within 36 h. Nitrogen-transformation-related enzyme activities were determined using an ELISA detection kit and an ELISA reader (Thermo Fisher). The organic matter was measured using the K_2_CrO_7_–H_2_SO_4_ oxidation–reduction titration method. A FlashEA 1112 series CHNS/O analyzer (Thermo Fisher, Waltham, MA, USA) was used to determine the total C (TC) and total P (TP) contents in the shoots, roots, and soils. To determine the plant dry weight, all the plant samples were oven-dried at 80 °C until they reached a constant weight (9). Ergonovine was detected using an Agilent ChemStation for GC−LC Rev.A.10.01 systems (Agilent Technologies, Santa Clara, CA, USA) [39]. The mycelium concentration of the *E. gansuensis* endophyte in *A. inebrians* was measured using quantitative polymerase chain reaction (QPCR) [40].

### 2.9. Statistical Analysis

All the statistical analyses were primarily performed with the R v.4.0.3 (http://www.r-project.org/) and IBM SPSS Statistics v.21 software. A normal distribution and the homogeneity of variances of the data were tested with the Shapiro–Wilk’s test and Levene’s test. The effect of the *Epichloë* endophyte and endophytic bacteria on the plant weight, tiller, plant nutrient uptake, and *nifH* gene abundance was assessed using a two-factor ANOVA followed by Tukey’s HSD test. If no significant interactions were detected between the *Epichloë* endophyte and the endophytic bacteria, significant differences in their individual effects on parameter variances were evaluated using an independent samples *t*-test. Indoleacetic acid was assessed for variation among the endophytic bacteria using a one-way analysis of variance (ANOVA) followed by Tukey’s HSD test. Spearman’s correlation coefficients were calculated among the plant weight, tiller, plant and soil nutrients, *nifH* gene abundance, ergonovine, and mycelium concentration to explore the relationship between the *Epichloë* endophyte and the endophytic bacteria and to determine whether their interactions could promote nitrogen transformation in the *A. inebrians* host.

## 3. Results

### 3.1. Community Structure and Function of Endophytic Bacteria in Roots of A. inebrians

**Figure 1:**
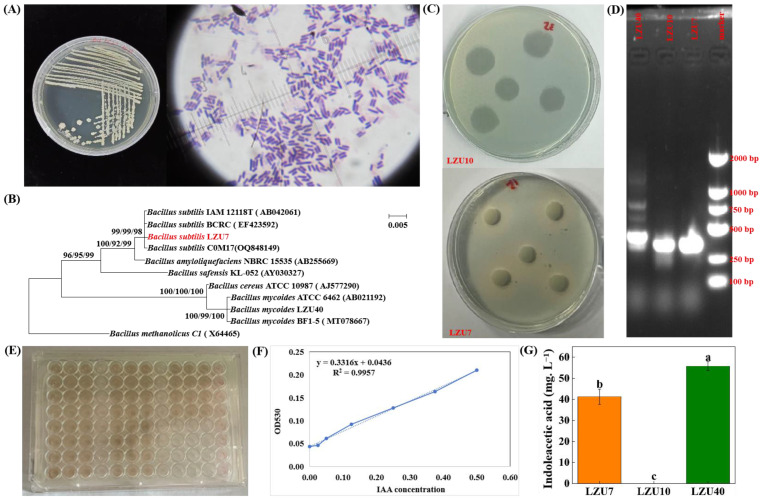
Morphological characterization and functional analysis of *Bacillus subtilis* LZU7. (**A**) Colony morphology, colony color, shape and size observations, and Gram staining. Purple indicates Gram-positive bacteria, while red indicates Gram-negative bacteria. (**B**) Determining genetic relationships among endophytic bacteria by building a phylogenetic tree based on the 16S rDNA sequences of the bacteria and its GenBank allies. (**C**) Ferritin analysis. Change in color indicates that the strain has the ability to secrete ferritin. (**D**) Gel electrophoresis map of the *nifH* gene in endophytic bacteria. The DNA fragment size of the *nifH* gene is 480 bps. The presence of a band at 480 bp indicates that the strain contains the *nifH* gene. (**E**) The determination of plant hormone indole-3-acetic acid. (**F**,**G**) The standard curve and concentration of indole-3-acetic acid. Values are means ± standard error. Soild and dotted lines: fitted and measured standard curves. Different lowercase letters indicate significant differences (*p* < 0.05) in the concentration among different strains.

We isolated and identified a total of 46 bacterial isolates from *A. inebrians* roots, and mainly focused on bacterial isolates belonging to the phylum Actinomycetota (1 isolate), Firmicutes (21 isolates), and Proteobacteria (24 isolates) (Table S1 and Figure S3). Five Proteobacteria and two Firmicutes isolates had the ability to secrete IAA, and seven Firmicutes and two Proteobacteria had the ability to produce siderophores (Table S1). In particular, the two tested Firmicutes isolates had the capacity to secrete growth hormones and siderophores, were able to grow in a nitrogen-free medium, and contained the *nifH* gene across all the tested isolates (Figure 1). However, the *E. gansuensis* endophyte strains had no growth-promoting effects (Figure S2).

### 3.2. E. gansuensis Endophyte Promotes the Growth and Colonization of Bacillus subtilis LZU7

**Figure 2:**
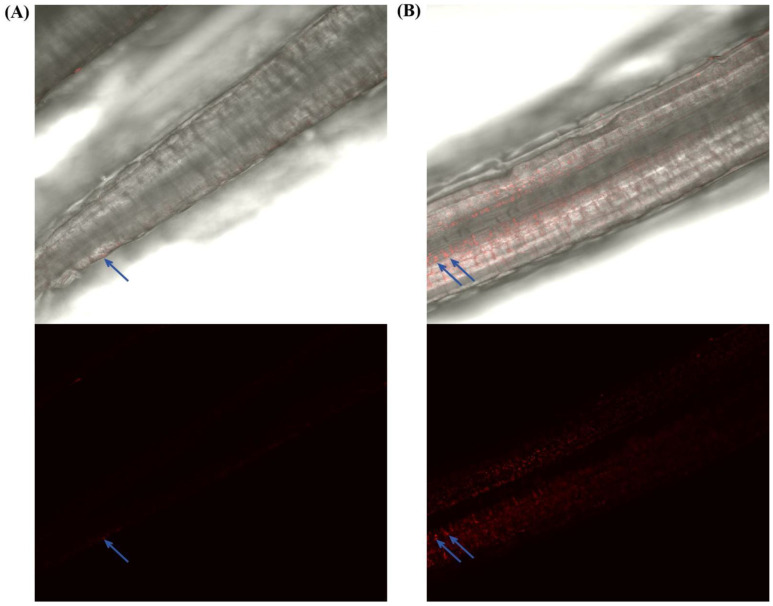
Colonization of GFP-tagged *Bacillus subtilis* LZU7 in *Achnatherum inebrians* roots. (**A**) Root tissues of endophyte-free (E−) plants. (**B**) Root tissues of endophyte-infected (E+) plants. Arrows indicate the presence of *B. subtilis* LZU7.

The total plate count of *B. subtilis* LZU7 and *B. mycoides* LZU40 from the phylum Firmicutes with a growth-promoting effect was measured using the plate confrontation method. Compared with the sterile water and PDA culture medium treatments, the fungal endophyte fluid significantly increased the number of *B. subtilis* LZU7 colonies, but there was no significant effect on the number of *B. mycoides* LZU40 colonies (Table 1). We observed no inhibition of the colony growth for *B. subtilis* LZU7 or *B. mycoides* LZU40 by the *E. gansuensis* endophyte (Figure S4). There was less GFP-tagged *B. subtilis* LZU7 in the roots when the plants were not infected with the *E. gansuensis* endophyte (Figure 2A). When plants were infected with the *E. gansuensis* endophyte, more GFP-tagged *B. subtilis* LZU7 was detected in the roots of *A. inebrians* (Figure 2B).

### 3.3. E. gansuensis Endophyte and B. subtilis LZU7 Promote A. inebrians Growth and Increase Yield

**Figure 3:**
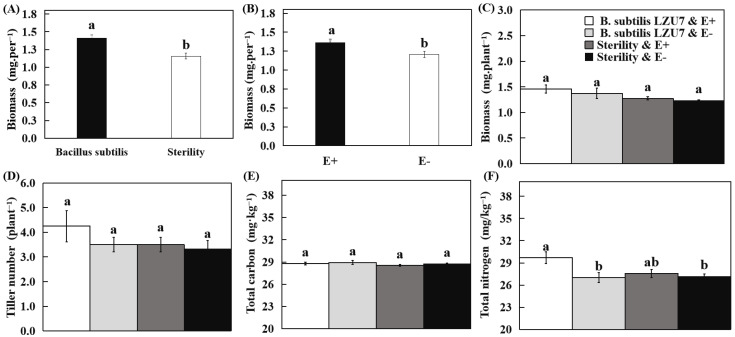
The effects of endophytic fungal (*Epichloë gansuensis*) and bacterial (*Bacillus subtilis LZU7*) interactions on plant performance metrics. (**A**) Biomass differences in plants grown in *B. subtilis* LZU7-inoculated versus non-inoculated soils. Different lowercase letters indicate significant differences (*p* < 0.05) between the inoculation and non-inoculation treatments according to Student’s *t*-test. (**B**) Biomass between endophyte-infected (E+) plants and endophyte-free (E−) plants. Different lowercase letters indicate significant differences (*p* < 0.05) between E+ and E− plants according to Student’s *t*-test. (**C**–**F**) Biomass, tiller number, total nitrogen, and total carbon between E+ and E− plants grown in inoculated and non-inoculated *B. subtilis* LZU7 soils. Different lowercase letters indicate significant difference (*p* < 0.05) by two-factor analysis of endophyte infection and *B. subtilis* inoculation.

The biomass of the plants inoculated with *B. subtilis* LZU7 reached 1.41 ± 0.20 mg, which was significantly higher than the biomass of the non-inoculated plants (1.16 ± 0.08) (*p* < 0.001) (Figure 3A). The biomass of the plants infected with the *E. gansuensis* endophyte was 1.37 ± 0.11 mg, which was significantly greater than that of non-infected plants (1.20 ± 0.01 mg) (*p* < 0.05) (Figure 3B). However, no significant effects on plant biomass were observed due to the interaction between an endophyte infection and *B. subtilis* LZU7 inoculation (Figure 3C). The results indicated that neither an endophyte infection nor *B. subtilis* LZU7 inoculation significantly promoted tillering and total carbon (Figure 3D,E). (Figure 3C). Total nitrogen was significantly higher in E+ plants (29.69 ± 0.87 mg. g^−1^) under the *B. subtilis* LZU7 inoculation treatment than in E+ (27.01 ± 0.67 mg. g^−1^) and E− (27.57 ± 0.54 mg. g^−1^) plants under the non-inoculation treatment, and in E− (27.18 ± 0.32 mg. g^−1^) plants under the inoculation treatment (Figure 3D).

### 3.4. Interactions Between E. gansuensis and B. subtilis LZU7 Enhance Nitrogen Fixation in A. inebrians

**Figure 4:**
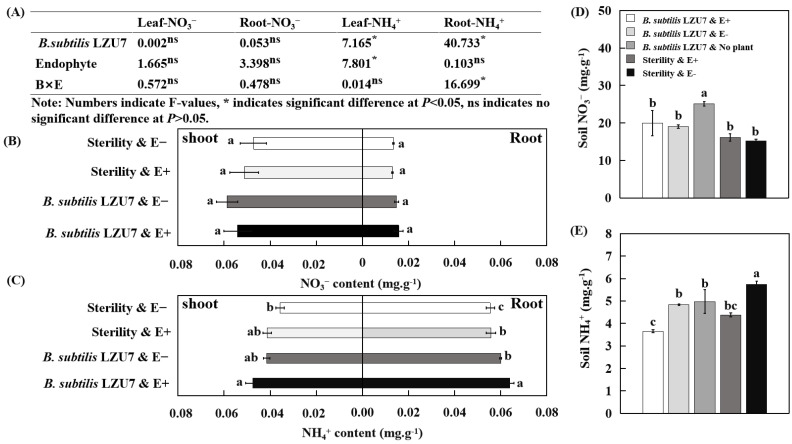
Changes in the NH_4_^+^ content in the leaves, roots, and soil due to an *E. gansuensis* infection and *B. subtilis* LZU7 inoculation. (**A**) A two-factor analysis of the effects of *E. gansuensis* and *B. subtilis* LZU7 on the NH_4_^+^ and NO_3_^−^ contents. (**B**,**C**) The concentration of NH_4_^+^ and NO_3_^−^ in the leaves and roots between E+ and E− plants grown in inoculated and non-inoculated *B. subtilis* LZU7 soils. The left side represents the leaf content, while the right side represents the root content. (**D**,**E**) The soil NH_4_^+^ and NO_3_^−^ concentrations after planting E+ and E− plants in inoculated and non-inoculated *B. subtilis* LZU7 soils. Different lowercase letters indicate significant differences between treatments (*p* <  0.05).

The leaf NH_4_^+^ content of E+ plants under the *B. subtilis* LZU7 inoculation condition were found to be much higher than in E− plants under the *B. subtilis* LZU7 non-inoculation condition, while the plants infected with *E. gansuensis* endophyte under the non-inoculation condition and non-infected plants under the inoculation condition showed no significant differences (Figure 4A,C). For root NH_4_^+^, the *E. gansuensis* endophyte-infected plants under the *B. subtilis* LZU7 inoculation condition showed a significantly higher content than the E− plants under inoculation or non-inoculation conditions, and the E+ plants under the non-inoculation condition (Figure 4A,C). The inoculation of *B. subtilis* LZU7 and its combination with an endophyte infection did not change the NO_3_^−^ content in the leaves, roots, or soil of *A. inebrians* (Figure 4 A,B,D). However, these treatments had a strong effect on the soil NH_4_^+^ content (Figure 4E).

For the inoculation treatment with *B. subtilis* LZU7, the root nitrogenase activity was significantly greater in E+ plants than in E− plants, while it was not significantly affected by an endophyte infection under the non-inoculation treatment (Figure S5A). The *E. gansuensis* endophyte infection significantly increased the nitrate reductase activity in the roots of *A. inebrians* (Figure S5B). The nitrate reductase and nitrite reductase activities in the roots of *A. inebrians* plants grown in soil inoculated with *B. subtilis* LZU7 were significantly greater than that in the roots of *A. inebrians* plants grown in soil without inoculated *B. subtilis* LZU7 soil (Figure S5C,D). Likewise, the glutamine synthetase activity in the roots was significantly (*p* < 0.05) greater in E+ plants compared with E− plants (Figure S5E), despite the fact that this activity in E+ and E− plants was not significantly affected by inoculation with *B. subtilis* LZU7. However, four enzymatic activities in the roots were not significantly affected by the interactions between the *E. gansuensis* infection and the *B. subtilis* LZU7 inoculation.

When the soil was not inoculated with *B. subtilis* LZU7, the CO_2_ and CH_4_ fluxes did not change, regardless of *E. gansuensis* infection. When the soil was inoculated with *B. subtilis* LZU7, the CO_2_ and CH_4_ fluxes were significantly (*p* < 0.05) increased (Figure S6A,B). The CO_2_ and CH_4_ fluxes were significantly increased when planting in soil inoculated with *B. subtilis* LZU7 compared with planting in soil that was not inoculated with *B. subtilis* LZU7; however, they were not significantly affected by the presence of the *E. gansuensis* endophyte (Figure S6A,B). The N_2_O flux did not change significantly between soils planted with E+ and E− plants under the conditions of the inoculation or non-inoculation with *B. subtilis* LZU7. However, there was a significant difference in the N_2_O flux between the soils inoculated and non-inoculated with *B. subtilis* LZU7 (Figure S6C).

**Figure 5:**
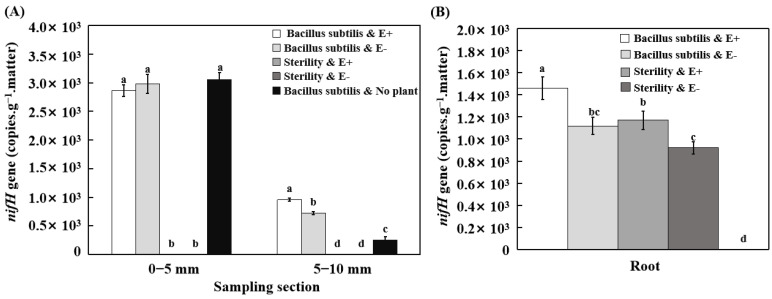
Potential nitrogen-fixing ability of roots and soil by *E. gansuensis* infection and *B. subtilis* LZU7 inoculation. (**A**) The copy number of the *nifH* gene in soil. (**B**) The copy number of *nifH* gene in roots of E+ and E− plants. Different lowercase letters indicate significant differences between treatments (*p*  <  0.05).

In the 0–5 cm soil layer, the *nifH* gene copy number clearly increased, with a significantly higher nifH gene copy number in soil inoculated with *B. subtilis* LZU7 than in soil non-inoculated with *B. subtilis* LZU7. However, the soil *nifH* gene copy number was not increased by endophyte-infected plants compared with endophyte-free plants, under both the inoculated and non-inoculated treatments (Figure 5A). In the 5–10 cm soil layer, the treatment inoculated with *B. subtilis* LZU7 also had an increased soil *nifH* gene copy number compared with the treatment not inoculated with *B. subtilis* LZU7. Furthermore, the nifH gene copy number was significantly higher in the soil with E+ plants than in the soil with E− plants under the treatment inoculated with *B. subtilis* LZU7 (Figure 5A). In the roots, the interactions between an *E. gansuensis* infection and *B. subtilis* LZU7 inoculation significantly increased the *nifH* gene copy number (Figure 5B).

**Figure 6:**
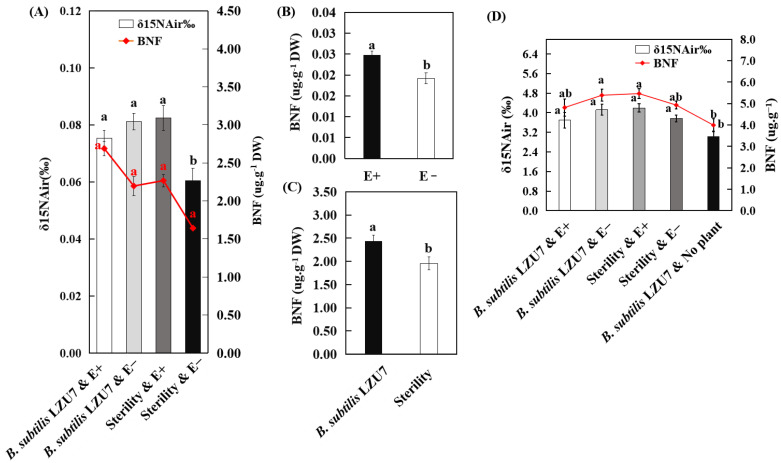
The effects of *E. gansuensis* endophyte infection and *B. subtilis* LZU7 inoculation on δ^15^N signatures and biological nitrogen fixation (BNF) in plant–soil systems. (**A**) δ ^15^N and biological nitrogen fixation (BNF) between E+ and E− plants grown in inoculated and non-inoculated *B. subtilis* LZU7 soils. Bar graph represents δ ^15^N. Red polyline represents BNF. (**B**) BNF between E+ and E− plants. Different lowercase letters indicate significant difference (*p* < 0.05) between E+ and E− plants by Student’s *t*-test. (**C**) Plant BNF between inoculated and non-inoculated *B. subtilis* LZU7 treatments. (**D**) Soil δ *^15^*N and biological nitrogen fixation.

The δ ^15^N of the plants was impacted by an *E. gansuensis* infection and *B. subtilis* LZU7 inoculation, with a significant increase in the environments where the E+ and E− plants grew under the treatment of *B. subtilis* LZU7 inoculation, and in the environments where the E+ plants grew under non-inoculation conditions (Figure 6A). The presence of the *E. gansuensis* endophyte resulted in significantly increased plant BNF, which was 0.03 ± 0.005 μg·g^−1^ DW, compared with the endophyte-free plants, with a content of 0.019 ± 0.008 μg·g^−1^ DW (Figure 6B). The plant BNF significantly increased by 24.5% in response to *B. subtilis* LZU7 inoculation (Figure 6C). However, the combined cultivation of E+ plants and inoculation with *B. subtilis* LZU7 did not lead to a significant effect on the concentration of δ ^15^N or BNF in the soil (Figure 6D).

### 3.5. E. gansuensis Endophyte Increased the Nitrogen and Biomass Accumulation of A. inebrians by Interacting with B. subtilis LZU7

**Figure 7:**
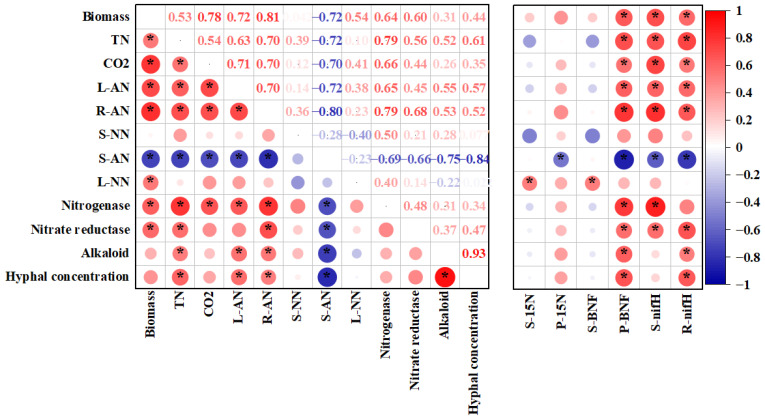
Correlation analysis among plant biomass, nutrients, greenhouse gases, nitrogen-conversion-related enzymes, hyphal concentration, alkaloid, nifH gene, δ ^15^N, and BNF. * Indicates significant correlations. The size of positive and negative numbers indicates the positive and negative correlation coefficients. Red circles indicate a positive correlation, while blue circles indicate a negative correlation. The larger the circle, the stronger the correlation between both. TN: plant total N content; CO_2_: CO_2_ flux; L-AN: leaf NH_4_^+^ content; R-AN: root NH_4_^+^ content; S-NN: soil NO_3_^−^ content; S-AN: soil NH_4_^+^ content; L-NN: leaf NO_3_^−^ content; P-BNF and S-BNF: biological nitrogen fixation in plant and soil; R-*nifH* and S-*nifH*: the copy number of the *nifH* gene in root and soil samples; P-15N and S-15N: the concentration of δ ^15^N in plant and soil.

We observed that the copy number of the *nifH* gene in the roots and the plant BNF were significantly and positively correlated with the biomass; the concentration of TN in the plant; the CO_2_ flux; the NH_4_^+^ content in the leaves and roots; and the nitrate reductase, alkaloid, and hyphal concentrations, whereas a negative association was observed between the plant BNF and the soil NH_4_^+^ content (*p* < 0.05) (Figure 7). Moreover, the plant BNF showed significant positive correlations with nitrogenase (Figure 7). The alkaloid and hyphal concentrations showed a better correlation with plant BNF (Figure 7), even though there was no statistically significant difference in the E+ plants under the *B. subtilis* LZU7 inoculation treatment (Figure S7). No significant correlations were detected between the content of plant ^15^N and other variables, except for a negative correlation with the soil NH_4_^+^ content (Figure 7). The interaction of the *E. gansuensis* endophyte with *B. subtilis* LZU7 increased the NH_4_^+^ accumulation in the leaves and roots compared with the control, which showed positive correlations with the biomass, plant TN, CO_2_ flux, *nifH* gene copy number in the leaves and roots, and plant BNF (*p* < 0.05) (Figure 7). Furthermore, we observed a positive relationship between biomass accumulation and the plant TN, the NH_4_^+^ in the leaves and roots, the CO_2_ flux, the leaf NO_3_^−^, nitrogenase, and nitrate reductase, but a strong negative correlation between biomass accumulation and the soil NH_4_^+^ content (Figure 7).

**Figure 8:**
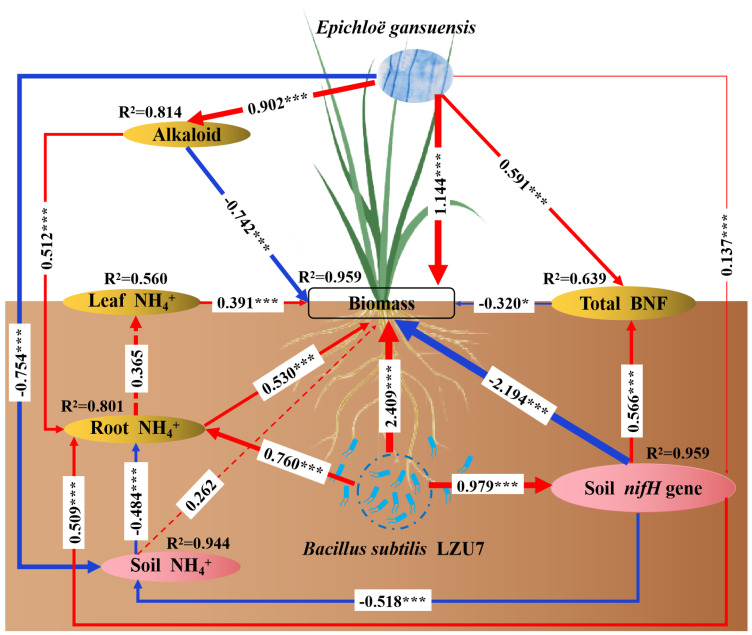
Structural equation modeling (SEM) shows the potential pathways that the interactions of *E. gansuensis* infection and *B. subtilis* LZU7 inoculation enhance plant performance. Overall, 4 treatments (*B. subtilis* LZU7 and E+, *B. subtilis* and E−, sterility and E+, and sterility and E−) with 4 replicates per treatment in the glasshouse experiment (16 datapoints) were included in this model. In this model, we quantified endophyte infection and *B. subtilis* LZU7 as their detected number. NH_4_^+^ content in leaves, roots, and soil, total BNF, soil *nifH* gene, and alkaloid were quantified as their response variables. *A. inebrians* performance was quantified as the variable of plant dry biomass. Red arrows indicate a positive correlation; blue arrows indicate a negative correlation. In the model, numbers on arrows are standardized path coefficients (SPC), indicating the strength of the relationship (* *p* < 0.05, and *** *p* < 0.001). The width of arrows is proportional to the strength of path coefficients. The numbers (R^2^) on the top of the response variables represent the proportion of explained variance. Results of model fitting: Chi-square (χ^2^) = 7.150, degrees of freedom (df) = 13, and probability level (*p*) = 0.894.

We used SEM to decipher how an endophyte infection affects plant performance by interacting with *B. subtilis* LZU7 (Figure 8). The SEM results indicated that the *E. gansuensis* endophyte had positive effects on plant performance by recruiting *B. subtilis* LZU7 colonization to increase the root N accumulation (Figure 8). The *E. gansuensis* endophyte and *B. subtilis* LZU7 had direct effects on plant biomass. Furthermore, changes in the soil *nifH* gene mediated by the *E. gansuensis* endophyte and *B. subtilis* LZU7 further affected the soil NH_4_^+^ content and led to a change in the root NH_4_^+^ content, and, thus, increased biomass accumulation (Figure 8). The root NH_4_^+^ content and the soil *nifH* gene were the main factors affecting plant biomass (Figure 8).

## 4. Discussion

The symbiotic fungal endophyte of *A. inebrians*, the *E. gansuensis* endophyte, has been found to affect the composition of root-associated bacteria by mediating root metabolism, thereby resulting in a positive response to low-nitrogen stress [29]. In this study, we found that the foliar *E. gansuensis* endophyte promoted the colonization of *A. inebrians* roots by root-originated *B. subtilis* LZU7, resulting in an increase in the N accumulation and biomass of the host plants.

Initially, we studied whether inhibition was present between the *E. gansuensis* endophyte strains and the *B. subtilis* LZU7 and *B. mycoides* LZU40 strains under in vitro culture conditions. The strains of the *E. gansuensis* endophyte lacked the ability to secrete IAA and siderophores and possessed no nitrogen fixation gene (*nifH* gene) (Figure S2), but the *B. subtilis* LZU7 and *B. mycoides* LZU40 strains had these functions (Figure 1). The effect of the *E. gansuensis* endophyte on the growth of *B. subtilis* LZU7 and *B. mycoides* LZU40 was explored from many angles. First, we used a solid medium to test the inhibition between both strains. The results showed that there was no inhibitory effect between the *E. gansuensis* endophyte and *B. subtilis* LZU7 or *B. mycoides* LZU40, but a growth-promoting effect was not obvious. Second, the fermentation broth of *E. gansuensis* endophyte strains increased the number of *B. subtilis* LZU7 colonies, but had no effect on *B. mycoides* LZU40, suggesting that the *E. gansuensis* endophyte has the potential to improve the growth of LZU7 strains. However, the strains and species used are taxonomically diverse and possibly metabolically different. The probiotic substances secreted by the *Epichloë* endophyte should be considered for further study.

Second, we demonstrated that an *E. gansuensis* endophyte infection could promote the colonization of A. inebrians roots by *B. subtilis* LZU7. We found that a large amount of GFP-tagged *B. subtilis* LZU7 aggregated in the roots of the symbionts of *E. gansuensis*–*A. inebrians* (Figure 2). These results suggest that the *Epichloë* endophyte plays an important role in the recruitment of beneficial microbes. *Epichloë* endophyte-induced changes and the enrichment of beneficial microbes were mainly attributed to two mechanisms related to altering the host’s metabolism. First, the endophytic-fungal-mediated metabolites of host plants could change the bacterial community composition directly [41,42], as symbiotic microorganisms have been shown to exhibit metabolic differences between different symbionts. For example, the *E. gansuensis* endophyte has been shown to mediate the production of secondary metabolite products, while alkaloids produced by *A. inebrians* could increase the colonization of beneficial root microbiomes and increase the complexity of interactions [29]. However, a change in beneficial microbes was inconsistent with the results of Rojas et al. [43], which showed that the infection of *Schedonorus arundinaceus* with *E. coenophiala* leads to a reduction in arbuscular mycorrhizal colonization. This suggests that the effects of an *Epichloë* endophyte infection on beneficial microbes may vary depending on the endophyte and/or host species.

Positive effects of the *Epichloë* endophyte on plant growth, fitness, defense against pathogens, tolerance to drought stress, and herbivore resistance have been documented [14,44,45,46], but the effect of its interactions with beneficial root microbes on the productivity and nutrient absorption of host plants is unknown. Zhao et al. [35] reported that the interaction between the *E. gansuensis* endophyte and Bacillus strains increased seed germination and the growth of *A. inebrians*. This increase might result from secondary metabolites from probiotic metabolism [47]. Our study demonstrates that productivity and nutrient absorption are also affected by the *E. gansuensis* endophyte and *B. subtilis* LZU7. Contrary to Zhao et al. [35], we observed that endophyte- or rhizobacteria-enhanced plant growth manifested as increased host biomass; *E. gansuensis* endophyte-infected plants or plants grown in soil inoculated with *B. subtilis* LZU7 gained more plant biomass compared with *E. gansuensis*-free plants or plants grown in soil not inoculated with *B. subtilis* LZU7, but tillers were not affected by an *E. gansuensis* endophyte infection or *B. subtilis* LZU7 inoculation (Figure 3). Therefore, the foliar *E. gansuensis* endophyte and root *B. subtilis* can improve plant productivity in addition to promoting seed germination.

The effects of the *E. gansuensis* endophyte on the nitrogen uptake efficiency in host plants and on soil nitrogen and phosphorus cycling may depend on plant enzyme activities and beneficial rhizobacteria interactions [30,48,49]. Importantly, we demonstrated that the root NH_4_^+^ concentrations were significantly increased for *E. gansuensis* endophyte-infected plants grown in soil inoculated with *B. subtilis* LZU7 compared with *E. gansuensis* endophyte-infected plants grown in non-inoculated soils and *E. gansuensis* endophyte-free plants grown in inoculated and non-inoculated soils (Figure 4C). The root NH_4_^+^ concentration had a significantly positive association with plant nitrogenase and nitrate reductase activities and with the *nifH* gene copy number in the roots and soil (Figure 7), suggesting that the interactions between the *E. gansuensis* endophyte and *B. subtilis* LZU7 promote nitrogen fixation and transformation; this is also supported by the significantly high enzyme activity and BNF (Figure 5 and Figure 6 and S5).

The synthesis of large amounts of alkaloids was associated with the depletion of plant N, in accordance with previous studies (31, 32) in which no significant differences were observed in the leaf NH_4_^+^ concentration between E+ and E− plants grown in inoculated or non-inoculated soils, but higher leaf NH_4_^+^ concentrations were observed for E+ plants grown in soils inoculated with *B. subtilis* LZU7 relative to E− plants grown in soils not inoculated with *B. subtilis* LZU7. Ren et al. [50] found that an endophyte infection tended to reduce the shoot nitrogen (N) concentration but caused a significant increase in the fractions of N allocated by the host plants. Furthermore, we found 62.31 mg.kg^−1^ and 59.06 mg.kg^−1^ of ergot alkaloids in E+ plants grown in soils inoculated and not inoculated with *B. subtilis* LZU7, respectively (Figure S7). Both of these ergot alkaloid concentrations increased significantly in response to an endophyte infection in interactive systems (Figure 8). These results indicate that an endophyte infection can promote nitrogen uptake and utilization efficiency in host plants.

The interactions of the *E. gansuensis* endophyte with *B. subtilis* LZU7 will change the soil NH_4_^+^ concentration, which will alter the root NH_4_^+^ concentration and lead to a biomass shift (Figure 8). Under low-nutrient conditions, an endophyte infection could be used to increase the root micro-nutrient content (e.g., K and Ca) to promote biomass accumulation and alleviate nutrient starvation stress (9). This tripartite interaction involving foliar fungal endophytes, host plants, and root bacteria can stimulate nitrogen-fixing microorganism activity, thereby improving the N bioavailability by decreasing molecular N and increasing BNF (Figure 4, Figure 5, Figure 6 and Figure 7). This process not only conducts N fixation, but also N transformation, leading to positive feedback between both. The relationship among endophyte infections, the nutrient availability, N acquisition, and biomass accumulation could be negative or positive. We also found that the interaction between the *E. gansuensis* endophyte and *B. subtilis* LZU7 increased the soil *nifH* gene copy number, which in turn affected the NH4+ content in the roots and soil and ultimately led to an increase in biomass (Figure 8). As previously reported, a low soil nutrient condition is suggested to benefit N acquisition and biomass accumulation by stimulating the effects of endophyte infection in enhancing cooperation and interactions among root bacteria [10,29]. However, there is also a contrasting perspective that high nutrient levels provide a greater opportunity for host plants to thrive, leading to positive correlations among nutrient resource availability, endophyte infections, and biomass accumulation [51,52]. This is because the *Epichloë* endophyte is a heterotrophic microorganism that competes with the host for nutrients or photosynthate at a metabolic cost at a low nutrient level [6,53]. Taken together, changes in the nutrient contents in the plants and soil were confirmed to contribute significantly to plant N acquisition and growth mediated by endophyte infections. This implies that *E. gansuensis* endophyte interactions with *B. subtilis* LZU7 are intricate at the interface between above-ground and below-ground plant parts. We, therefore, conclude that nutrient fraction connections driven by the *E. gansuensis* endophyte and *B. subtilis* LZU7 among different tissues is a pathway as important as the positive feedback between fungal endophyte interactions with *B. subtilis* LZU7 in enhancing plant performance.

## 5. Conclusions

This study demonstrates that the *E. gansuensis* endophyte can promote the growth of the plant growth-promoting rhizobacterium *B. subtilis* LZU7 in an in vitro culture and enhance its colonization in the roots of *A. inebrians* plants. We also confirmed that the *E. gansuensis* endophyte interactions with *B. subtilis* LZU7 had positive effects on nitrogenase (*nifH*) gene expression, which could lead to an increase in biological nitrogen fixation (BNF). These results also suggest that *E. gansuensis* infection and *B. subtilis* LZU7 inoculation regulate the interaction with nutrient fractions leading to biomass accumulation. The root NH_4_^+^ concentration was positively correlated with both *E. gansuensis* endophyte infection and *B. subtilis* LZU7 inoculation, and eventually improved the production of *A. inebrians*. This, in turn, led to negative feedback with soil N accumulation through affecting the copy number of the soil *nifH* gene, leading to a further increase in the root NH_4_^+^ content, and enhanced biomass accumulation. Our results provide a novel demonstration that interactions between the foliar *Epichloë* endophyte and the beneficial root-originated bacterium *B. subtilis* can have major effects on the uptake and utilization of an essential nutrient, and they suggest that the source of N resources can be from biological nitrogen fixation, which is mediated by nitrogen-fixing microorganisms.

## Figures and Tables

**Table 1:** The changes in total plate count of *B. Subtilis* LZU7 and *B. mycoides* LZU40 treated with fungal endophyte solution.

Bacteria Coating	Treatment	10^−5^	10^−6^
*B. subtilis* LZU7	Fungal endophyte fluid	59.25 ± 1.31 a	19.25 ± 3.98 a
	PDA culture medium	44.50 ± 3.88 b	12.00 ± 3.13 b
	Sterile water	44.50 ± 3.88 b	7.00 ± 0.91 bc
*B. mycoides* LZU40	Fungal endophyte fluid	36.00 ± 5.30 b	8.00 ± 0.91 bc
	PDA culture medium	40.25 ± 4.03 b	7.75 ± 0.63 bc
	Sterile water	19.75 ± 0.85 c	4.00 ± 250.85 c

Values are means ± standard error. Different lowercase letters indicate significant difference (*p* < 0.05) among different treatments.

## Data Availability

The original contributions presented in this study are included in the article/Supplementary Materials. Further inquiries can be directed to the corresponding authors.

## Supplementary Materials

The following supporting information can be downloaded at: https://www.mdpi.com/article/10.3390/jof11070466/s1, Figure S1 Schematic representation (A) and physical pictures (B) of experimental set-up; Figure S2 Functional identification of *Epichloë gansuensis* endophyte strains. (A) The surface and reverse views of *E. gansuensis* endophyte strain. (B) Gel electrophoresis map of the *nifH* gene in *E. gansuensis* endophyte strains. (C) Siderophores measurement of *E. gansuensis* endophyte strains. (D) Promoting ability of *E. gansuensis* endophyte strains; Figure S3 The genetic relationships analysis among 46 strains endophytic bacterium by building the phylogenetic tree; Figure S4 Plate confrontation test of the *E. gansuensis* endophyte strains and endophytic bacteria. (A) *B. Subtilis* LZU7 strains. (B) *B. mycoides* LZU40; Figure S5 Effects of *E. gansuensis* endophyte infection and *B. Subtilis* LZU7 inoculation on nitrogenase, nitrate reductase, nitrite reductase and glutamine synthetase of *A. inebrians.* (A) The two-factor analysis of enzymatic activity. (B,E) The one-factor analysis of nitrate reductase and nitrite reductase between E+ and E- plants. (C,D) The one-factor analysis of nitrate reductase and glutamine synthetase between inoculated and non-inoculated *B. subtilis* LZU7; Figure S6 The effects of the *E. gansuensis* endophyte infection and *B. Subtilis* LZU7 inoculation influences soil fluxes of the greenhouse gases CO_2_, CH_4_ and N_2_O. (A) CO_2_ flux; (B) CH_4_ flux; (C) N_2_O flux; Figure S7 The effects of the *E. gansuensis* endophyte infection and *B. Subtilis* LZU7 inoculation on mycelial gene copy number and alkaloid content. (A) Mycelial gene copy number; (B) Alkaloid content; Table S1 Isolation, identification and functional characterization of endophytic bacteria in *A. inebrians*.
