# Supplementary material for: Foliar Epichloë gansuensis Endophyte and Root-Originated Bacillus subtilis LZU7 Increases Biomass Accumulation and Synergistically Improve Nitrogen Fixation in Achnatherum inebrians
Source: J Fungi (Basel). 2025 Jun 20;11(7):466. doi: 10.3390/jof11070466 (PMC12295881; doi:10.3390/jof11070466)
Supplement: Supplementary file 1 [file jof-11-00466-s001.zip › jof-3630840-supplementary.pdf]

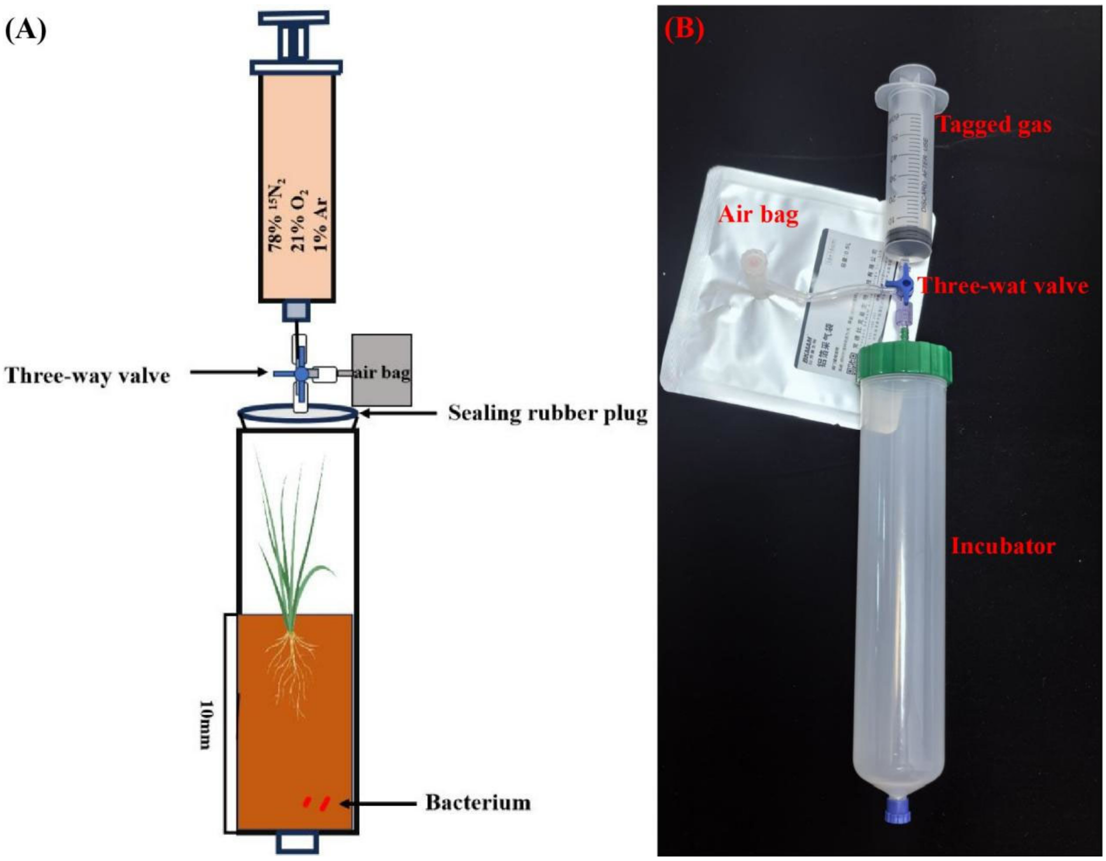

Figure S1. Schematic representation (A) and physical pictures (B) of experimental set-up.

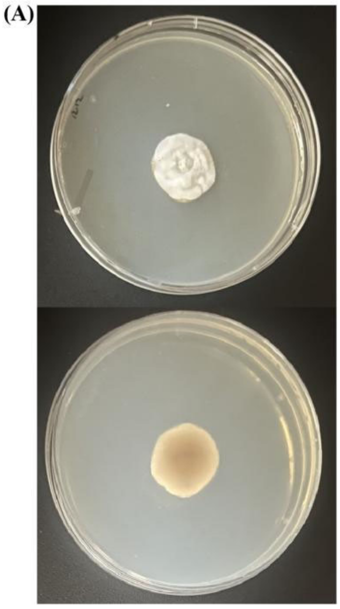

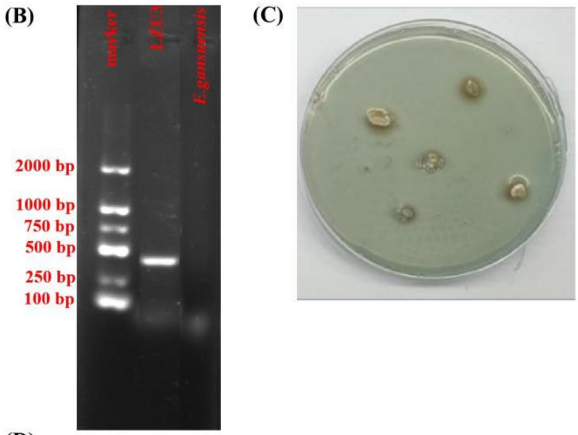

(D)

Table S1 Identification and growth promotion evaluation of *E. gansuensis* endophyte strains in the *Achnatherum inebrians* plants

Name	NCBI comparison	Carrier ferritin	Auxin (Acetic acid)	Growth without nitrogen medium	Denitrification
E. gansuensis	97.82%	-	-	-	-

Figure S2. Functional identification of *Epichloë gansuensis* endophyte strains. (A) The surface and reverse views of *E. gansuensis* endophyte strain. (B) Gel electrophoresis map of the *nifH* gene in *E. gansuensis* endophyte strains. (C) Siderophores measurement of *E. gansuensis* endophyte strains. (D) Promoting ability of *E. gansuensis* endophyte strains.

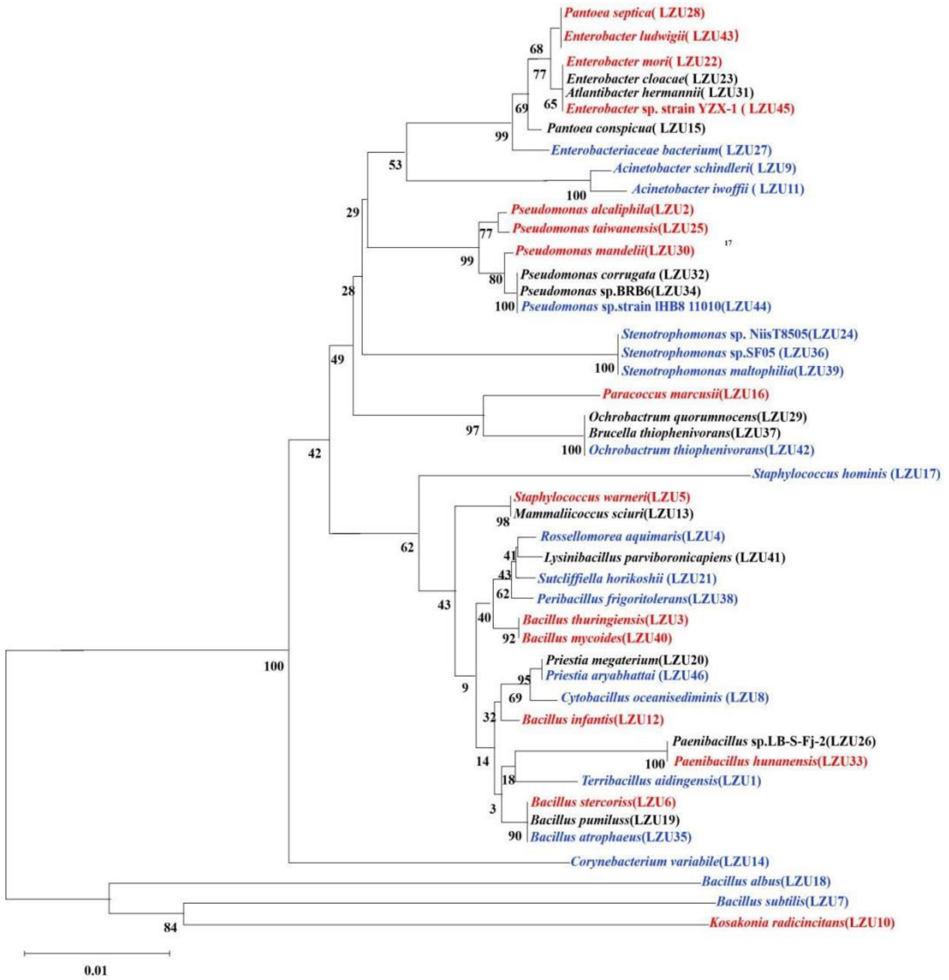

Figure S3. The genetic relationships analysis among 46 strains endophytic bacterium by building the phylogenetic tree.

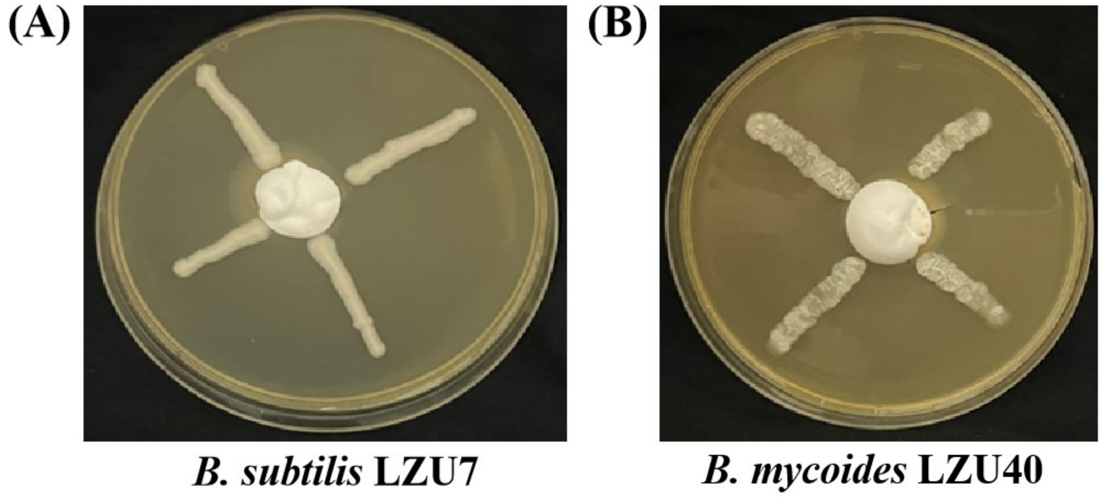

Figure S4 Plate confrontation test of the *E. gansuensis* endophyte strains and endophytic bacteria. (A) *B. Subtilis* LZU7 strains. (B) *B. mycoides* LZU40.

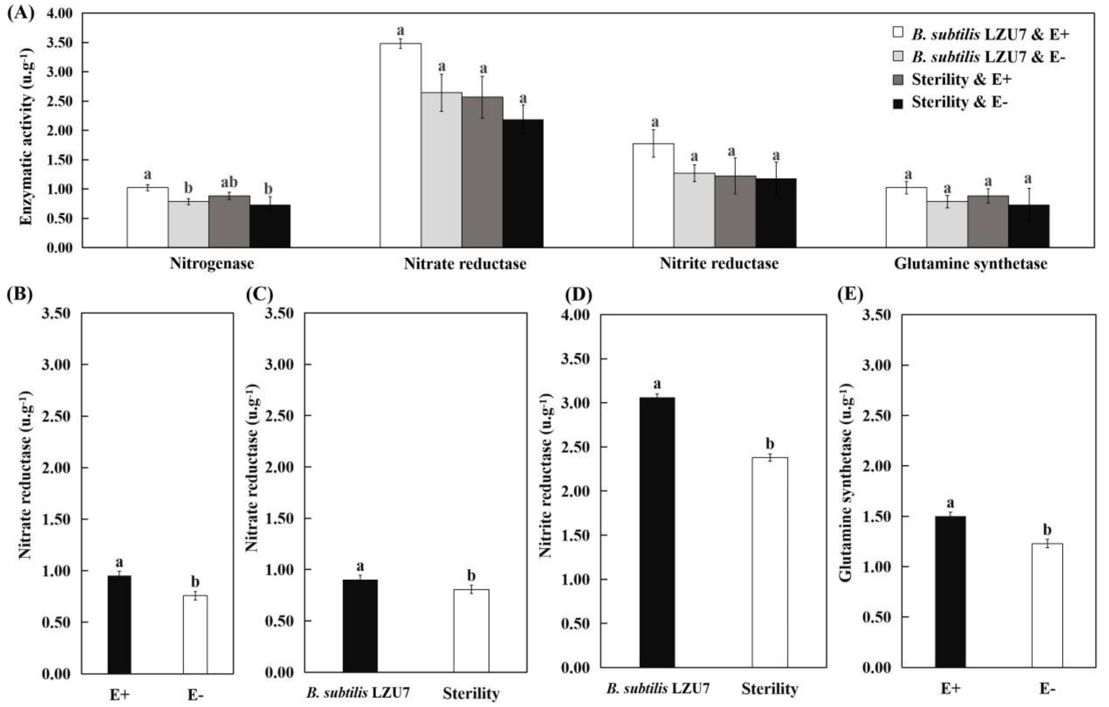

Figure S5 Effects of *E. gansuensis* endophyte infection and *B. Subtilis* LZU7 inoculation on nitrogenase, nitrate reductase, nitrite reductase and glutamine synthetase of *A. inebrians.* (A) The two-factor analysis of enzymatic activity. (B and E) The one-factor analysis of nitrate reductase and nitrite reductase between E+ and E- plants. (C and D) The one-factor analysis of nitrate reductase and glutamine synthetase between inoculated and non-inoculated *B. subtilis* LZU7.

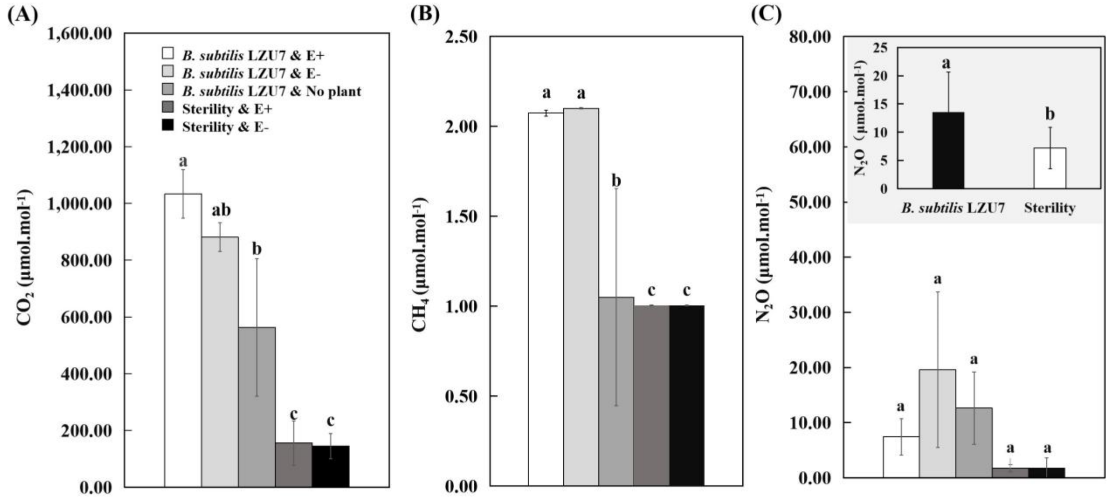

Figure S6 The effects of the *E. gansuensis* endophyte infection and *B. Subtilis* LZU7 inoculation influences soil fluxes of the greenhouse gases CO_2, CH_4 and N_2O. (A) CO_2 flux; (B) CH_4 flux; (C) N_2O flux.

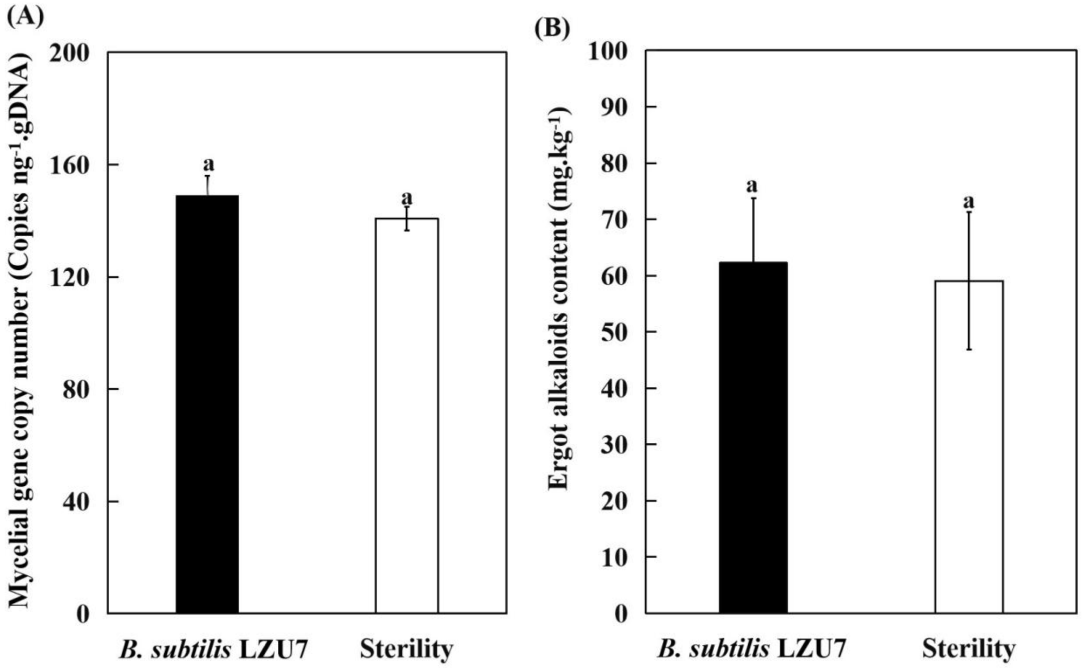

Figure S7 The effects of the E. gansuensis endophyte infection and B. Subtilis LZU7 inoculation on mycelial gene copy number and alkaloid content. (A) Mycelial gene copy number; (B) Alkaloid content.

Table S1. Isolation, identification and functional characterization of endophytic bacteria in *Achnatherum inebrians.*

Number	Name	Bacteria phylum	Bacteria species	Similarity	Gram test	Shape	Colony colour	Indoleacetic acid ($mg.L^{-1}$)	Transferrin	Nitrogen-free	*nifH* gene
1	LZU14	Actinomycetota	*Corynebacterium variabile*	99.78%	Negative	Round	Yellowish	-	-	-	-
2	LZU1	Firmicutes	*Terribacillus aidingensis*	99.86%	Positive	Rod	Yellowish	-	-	-	-
3	LZU3	Firmicutes	*Bacillus proteolyticus*	99.72%	Positive	Rod	White	-	-	-	-
4	LZU4	Firmicutes	*Rossellomorea aquimaris*	99.93%	Negative	Rod	Yellow	-	-	-	-
5	LZU5	Firmicutes	*Staphylococcus warneri*	99.93%	Positive	Round	White	-	-	-	-
6	LZU6	Firmicutes	*Bacillus stercoris*	99.86%	Negative	Rod	White	-	-	-	-
**7**	**LZU7**	**Firmicutes**	***Bacillus subtilis***	**99.26%**	**Positive**	**Rod**	**White**	**41.26**	**+**	**+**	**+**
8	LZU8	Firmicutes	*Cytobacillus oceanisediminis*	99.72%	Positive	Rod	Light pink	-	-	-	-
9	LZU12	Firmicutes	*Bacillus infantis*	99.65%	Positive	Rod	Orange red	-	-	-	-
10	LZU13	Firmicutes	*Mammaliicoccus sciuri*	99.79%	Negative	Round	Yellow	-	-	-	-
11	LZU17	Firmicutes	*Staphylococcus hominis*	99.97%	Positive	Round	White	-	-	-	-
12	LZU18	Firmicutes	*Bacillus albus*	94.29%	Positive	Rod	Yellowish	-	-	-	-
13	LZU1	Firmicutes	*Bacillus pumilus*	99.58%	Positive	Rod	White	-	+	-	-

	9										
14	LZU20	Firmicutes	*Priestia megaterium*	99.93%	Positive	Rod	Yellowish	-	-	-	-
15	LZU21	Firmicutes	*Sutcliffiella horikoshii*	99.93%	Negative	Rod	White	-	-	-	-
16	LZU26	Firmicutes	*Paenibacillus sp.* LB-S-FJ-2	99.03%	Positive	Rod	Yellowish	-	-	-	-
17	LZU33	Firmicutes	*Paenibacillus hunanensis*	99.03%	Positive	Rod	Light beige brown	-	+	-	-
18	LZU35	Firmicutes	*Bacillus atrophaeus*	100.00%	Positive	Rod	Dark brown	-	+	-	-
19	LZU38	Firmicutes	*Peribacillus frigoritolerans*	99.86%	Negative	Rod	Yellowish	-	+	-	-
20	LZU40	Firmicutes	*Bacillus mycoides*	100.00%	Positive	Rod	Creamy white	55.84	+	+	+
21	LZU41	Firmicutes	*Lysinibacillus parviboronicapiens*	99.44%	Negative	Rod	Earthy yellow	-	-	-	-
22	LZU46	Firmicutes	*Priestia aryabhattai*	99.93%	Negative	Rod	Milky white	-	+	-	-
23	LZU2	Proteobacteria	*Pseudomonas alcaliphila*	99.93%	Negative	Rod	Grayish white	-	-	-	-
24	LZU9	Proteobacteria	*Acinetobacter schindleri*	99.50%	Negative	Rod	Yellow	-	-	-	-
25	LZU10	Proteobacteria	*Kosakonia radicincitans*	96.88%	Negative	Rod	Yellowish	-	-	-	+
26	LZU1	Proteobacteria	*Acinetobacter lwoffii*	99.86%	Negativ	Roun	White	-	-	-	-

	1				e	d					
27	LZU15	Proteobacteria	*Pantoea conspicua*	99.85%	Negative	Rod	Yellow	-	-	-	-
28	LZU16	Proteobacteria	*Paracoccus marcusii*	99.85%	Negative	Round	Saffron yellow	-	-	-	-
29	LZU22	Proteobacteria	*Enterobacter mori*	99.52%	Negative	Rod	Pink	-	-	-	-
30	LZU23	Proteobacteria	*Enterobacter cloacae*	99.76%	Negative	Rod	Yellow	-	-	-	-
31	LZU24	Proteobacteria	*Stenotrophomonas sp.* NIIST B 505	98.80%	Negative	Rod	White	-	-	-	-
32	LZU25	Proteobacteria	*Pseudomonas taiwanensis*	99.52%	Negative	Rod	White	-	-	-	-
33	LZU27	Proteobacteria	*Enterobacteriaceae bacterium*	99.52%	Negative	Rod	White	-	+	-	-
34	LZU28	Proteobacteria	*Pantoea septica*	99.73%	Negative	Rod	Yellow	-	-	-	-
35	LZU29	Proteobacteria	*Ochrobactrum quorumnocens*	99.78%	Negative	Rod	Yellowish	-	-	-	-
36	LZU30	Proteobacteria	*Pseudomonas mandelii*	100.00%	Negative	Rod	White	-	-	-	-
37	LZU31	Proteobacteria	*Atlantibacter hermannii*	99.72%	Positive	Rod	Yellow	-	-	-	-
38	LZU32	Proteobacteria	*Pseudomonas corrugata*	99.86%	Positive	Rod	Grayish yellow	-	-	-	-
39	LZU3	Proteobacteria	*Pseudomonas sp.* BRB6	100.00%	Negativ	Rod	White	-	-	-	-

	4				e						
40	LZU36	Proteobacteria	*Stenotrophomonas sp.* SF05	99.65%	Negative	Rod	Yellowish	-	-	-	-
41	LZU37	Proteobacteria	*Brucella thiophenivorans*	99.93%	Negative	Rod	Sickly white	33.12	-	-	-
42	LZU39	Proteobacteria	*Stenotrophomonas maltophilia*	99.80%	Negative	Rod	Yellowish	45.68	-	-	-
43	LZU42	Proteobacteria	*Ochrobactrum thiophenivorans*	99.93%	Negative	Rod	Sickly white	15.73	-	-	-
44	LZU43	Proteobacteria	*Enterobacter ludwigii*	99.93%	Positive	Rod	Yellow	45.68	+	-	-
45	LZU44	Proteobacteria	*Pseudomonas sp. strain IHBB 11010*	99.72%	Negative	Rod	Green yellow	-	-	-	-
46	LZU45	Proteobacteria	*Enterobacter sp. strain* YZX-1	99.71%	Negative	Rod	Yellowish	-	-	-	-

Note: "+" means the test is positive, "-" means the test is negative.
